# Effects of auricular therapy on pain and anxiety in patients undergoing hysteroscopic examination: A retrospective comparative study

**DOI:** 10.1097/MD.0000000000049378

**Published:** 2026-06-19

**Authors:** Weizhu Zhu, Anqi Zhou, Xianwen Jin

**Affiliations:** a Center for Reproductive Medicine, The Fourth Affiliated Hospital of School of Medicine, and International School of Medicine, International Institutes of Medicine, Zhejiang University, Yiwu, Zhejiang, China.

**Keywords:** anxiety, auricular therapy, hysteroscopic examination, local anesthesia, Numeric Rating Scale, perioperative pain, Self-Rating Anxiety Scale

## Abstract

Hysteroscopic examination is commonly used to evaluate suspected intrauterine abnormalities, but procedure-related pain and anxiety may reduce tolerance and patient satisfaction. Auricular therapy is a nonpharmacologic intervention that may improve perioperative symptom control. This retrospective comparative study included 142 women who underwent hysteroscopic examination between September 2024 and December 2025. According to perioperative management, patients were divided into 4 groups: nonanesthesia without auricular therapy (group 1, n = 34), nonanesthesia with auricular therapy (group 2, n = 37), local anesthesia without auricular therapy (group 3, n = 32), and local anesthesia with auricular therapy (group 4, n = 39). Pain was assessed using the Numeric Rating Scale, and anxiety was evaluated using the Self-Rating Anxiety Scale. Intraoperative indicators, adverse events, and satisfaction were also recorded. Intraoperative and 30-minute postoperative Numeric Rating Scale scores differed significantly among groups (*F* = 5.220, *P* = .002; *F* = 11.019, *P* < .001), with group 2 showing the lowest pain scores. Among nonanesthetized patients, auricular therapy was associated with lower postoperative pain than no auricular therapy (mean difference 1.161, 95% confidence interval 0.710–1.611; *P* < .001). Postoperative 24-hour Self-Rating Anxiety Scale scores also differed significantly (*F* = 52.328, *P* < .001), with the highest score in group 1 and the lowest in group 2. No significant between-group differences were found in heart rate, blood pressure, or adverse event rates (all *P* > .05). All adverse events were mild and transient. Satisfaction was high in all groups, and the highest proportion of “very satisfied” patients was observed in group 2 (*P* = .013). Auricular therapy was associated with reduced perioperative pain, lower postoperative anxiety, and higher satisfaction during hysteroscopic examination, particularly in the nonanesthesia setting.

## 1. Introduction

Hysteroscopic examination is a central procedure in contemporary gynecologic practice because it permits direct visualization of the endocervical canal and uterine cavity and supports the diagnosis of intrauterine pathology in women with infertility, abnormal uterine bleeding, recurrent implantation failure, and other suspected cavity disorders. Recent fertility-focused reviews and guideline updates have emphasized that hysteroscopy remains clinically important not only for overt structural lesions but also for subtle intracavitary abnormalities that may be missed by conventional imaging and may still impair endometrial receptivity or reproductive outcomes. In women with suspected uterine cavity disorders, recent World Health Organization recommendations support structured cavity assessment using imaging- and hysteroscopy-based approaches according to local resources and clinical indication, underscoring the continuing relevance of hysteroscopy in reproductive evaluation and management.^[1–3]^ Despite its diagnostic utility and minimally invasive profile, hysteroscopy is frequently accompanied by procedural pain and anticipatory anxiety. These symptoms are clinically relevant because they can reduce procedure tolerance, increase difficulty with uterine entry, interfere with operator performance, and negatively affect the overall patient experience. Recent observational studies have shown that severe pain during office hysteroscopy is associated with higher anxiety levels and longer procedure duration, while patient satisfaction studies have further indicated that pain and the quality of preprocedural information are major determinants of global satisfaction after outpatient hysteroscopy.^[4,5]^

Current pain-control strategies for hysteroscopy include oral analgesics, nonsteroidal anti-inflammatory drugs, cervical local anesthesia, and selected nonpharmacologic interventions. However, the analgesic benefit of pharmacologic approaches is variable across procedural settings, and anesthetic techniques may not fully address the emotional component of pain perception. Increasing attention has therefore been directed toward adjunctive, low-risk, nonpharmacologic modalities capable of attenuating both nociceptive and anxiety-related responses during short ambulatory procedures.^[6,7]^ Auricular therapy is one such candidate strategy. From a traditional Chinese medicine perspective, auricular stimulation is used to regulate functional balance and relieve pain and emotional distress; from a contemporary perioperative perspective, it may also serve as a structured supportive intervention. Recent evidence suggests that acupressure can reduce preoperative anxiety in adults undergoing elective procedures, while observational and interventional studies of auricular plaster therapy, auricular acupressure, and auricular neuromodulation have reported favorable effects on perioperative anxiety, postoperative pain, or recovery-related symptoms. Although these data are not specific to hysteroscopy, they support the plausibility of ear-based interventions as adjuncts in procedure-related symptom management.^[8–10]^

Auricular therapy represents a structured auricular intervention based on fixed point selection and repeated stimulation. However, evidence regarding its application during hysteroscopic examination remains limited. In this context, the present study was designed to further evaluate the potential clinical relevance of auricular therapy for perioperative symptom control in women undergoing hysteroscopy.

## 2. Methods

### 2.1. Study design

This was a retrospective comparative study conducted in the Department of Gynecology of the Fourth Affiliated Hospital, Zhejiang University School of Medicine. Clinical data of women who underwent hysteroscopic examination for the evaluation of suspected intrauterine abnormalities between September 2024 and December 2025 were reviewed. According to perioperative analgesic management and auricular care documented in the medical and nursing records, eligible patients were categorized into 4 groups: nonanesthesia without auricular therapy (group 1), nonanesthesia with auricular therapy (group 2), local anesthesia without auricular therapy (group 3), and local anesthesia with auricular therapy (group 4). The study protocol was approved by the institutional ethics committee. The study was conducted in accordance with the Declaration of Helsinki. Written informed consent for the clinical procedures was obtained from all patients, and the requirement for separate informed consent for this retrospective analysis was waived by the ethics committee.

Treatment allocation was not randomized. During routine clinical practice, patients were informed of available perioperative management options, including local anesthesia and auricular therapy. The final management strategy was determined through shared decision-making between patients and healthcare providers according to patient preference, expected tolerance, and clinical considerations. Therefore, group assignment reflected real-world clinical practice rather than protocol-driven allocation.

### 2.2. Inclusion and exclusion criteria

Inclusion criteria were as follows:

1. age 20 to 45 years;
2. underwent hysteroscopic examination for evaluation of suspected intrauterine abnormalities;
3. American Society of Anesthesiologists physical status I to II; and
4. complete perioperative clinical and nursing records, including pain and anxiety assessments.

Exclusion criteria were as follows:

1. contraindications to hysteroscopy;
2. allergy to auricular patch materials, including Vaccaria segetalis seeds or adhesive tape;
3. local ear lesions, such as ulceration, swelling, deformity, or skin damage, precluding auricular manipulation;
4. severe psychiatric or neurological disorders affecting assessment reliability;
5. severe cardiovascular, cerebrovascular, hepatic, or renal disease;
6. obvious preprocedural symptoms such as marked pain, nausea, vomiting, dizziness, or tinnitus; and
7. incomplete key data or inability to complete outcome evaluation.

### 2.3. Perioperative management

All patients received routine perioperative nursing care for hysteroscopic examination, including preprocedural education, verification of examination-related contraindications, instructions regarding fasting and bladder emptying, intraoperative positioning and monitoring, and postoperative observation and health education. In the nonanesthesia setting, patients in the routine-care group received an intramuscular injection of parecoxib sodium 40 mg approximately 30 minutes before the procedure, whereas patients in the auricular therapy group received the same basic management plus auricular therapy. In the local anesthesia setting, patients in the routine-care group received cervical local infiltration anesthesia with 2% lidocaine, with a total volume of 5 mL injected at the 3, 6, and 9 o’clock positions of the cervix approximately 10 minutes before hysteroscopy. Patients in the local anesthesia plus auricular therapy group additionally received auricular therapy on the basis of local anesthesia and routine perioperative care. For patients who did not receive auricular therapy, placebo ear patches without Vaccaria segetalis seeds were applied, as documented in the nursing records.

### 2.4. Auricular therapy

Auricular therapy was administered by trained nurses in accordance with the national standard Standardized Manipulations of Acupuncture and Moxibustion – Part 3: Ear Acupuncture (GB/T 21709.3-2021). After disinfection of both auricles with 75% alcohol, specialized auricular patches containing Vaccaria segetalis seeds were applied bilaterally to the shenmen, pelvis, abdomen, and endocrine auricular points. Manual pressure was then applied alternately using the thumb and index finger, with the stimulation intensity gradually increased until the patient reported sensations such as soreness, numbness, distension, warmth, or mild pain. The first treatment session was initiated 15 minutes before the procedure and was performed in a fixed sequence of abdomen–shenmen–pelvis–endocrine. Each auricular point was pressed 30 times for 3 cycles, with a total treatment duration of approximately 10 minutes. The second treatment session was performed at the time of hysteroscope insertion, and the third session was completed immediately after the procedure. Patients were instructed to retain the auricular patches and perform self-pressing for 1 to 2 minutes per session, repeated 2 to 3 times within 24 hours after the procedure.

### 2.5. Outcome measures

Baseline data included age, body mass index, obstetric history, history of intrauterine manipulation, history of hysteroscopic surgery, and operative duration. Pain intensity was assessed using the Numeric Rating Scale (NRS), with scores ranging from 0 to 10, where higher scores indicated greater pain severity. NRS scores were recorded at vaginal disinfection, hysteroscope insertion, intraoperative manipulation, completion of the procedure, and 30 minutes after the procedure. Anxiety status was evaluated using the Self-Rating Anxiety Scale (SAS). SAS scores were assessed 30 minutes before the procedure and 24 hours after the procedure. The anxiety improvement value was calculated as the preprocedural SAS standard score minus the postoperative SAS standard score. Intraoperative variables included operative duration, hysteroscope placement time, heart rate, systolic blood pressure, and diastolic blood pressure. Adverse events occurring from the perioperative period to 24 hours postoperatively were also recorded, including nausea, vomiting, dizziness, cervical bleeding, vagal reactions, tinnitus, and auricular skin reactions. Patient satisfaction at 24 hours after the procedure was assessed using a self-developed nursing satisfaction questionnaire.

### 2.6. Statistical analysis

Statistical analysis was performed using SPSS version 31.0 (IBM Corp., Armonk, NY). Continuous variables with a normal distribution were expressed as mean ± standard deviation and compared using 1-way analysis of variance, followed by the least significant difference test for pairwise comparisons. Continuous variables with a nonnormal distribution were presented as median (interquartile range) and compared using the Kruskal–Wallis *H* test. Categorical variables were expressed as number (percentage) and compared using the chi-square test or Fisher exact test, as appropriate. A 2-sided *P* value < .05 was considered statistically significant.

## 3. Results

### 3.1. Baseline characteristics

According to perioperative management, 34 patients were assigned to group 1 (nonanesthesia without auricular therapy), 37 to group 2 (nonanesthesia with auricular therapy), 32 to group 3 (local anesthesia without auricular therapy), and 39 to group 4 (local anesthesia with auricular therapy). Comparisons of baseline variables showed no statistically significant differences among the 4 groups with respect to age, body mass index, history of childbirth, history of intrauterine manipulation, or procedure duration (all *P* > .05), indicating acceptable baseline comparability (Table 1).

**Table 1:** Baseline characteristics of patients undergoing hysteroscopic examination in the 4 groups.

Variable	Group 1: nonanesthesia without auricular therapy (n = 34)	Group 2: nonanesthesia with auricular therapy (n = 37)	Group 3: local anesthesia without auricular therapy (n = 32)	Group 4: local anesthesia with auricular therapy (n = 39)	*P* value
Age (yr)	33.12 ± 5.56	33.45 ± 5.40	32.85 ± 5.69	33.28 ± 5.50	.974
BMI (kg/m^2^)	23.09 ± 3.33	22.06 ± 2.75	22.35 ± 2.79	22.31 ± 2.49	.470
History of childbirth, n (%)	12 (35.29)	8 (21.62)	9 (28.12)	7 (17.95)	.347
History of intrauterine manipulation, n (%)	29 (85.29)	32 (86.49)	28 (87.50)	26 (66.67)	.065
Procedure duration (min)	11.84 ± 4.70	13.96 ± 4.80	12.79 ± 4.86	11.66 ± 3.87	.120

BMI = body mass index.

### 3.2. Comparison of NRS pain scores among the 4 groups

All patients had a preprocedural NRS score of 0. The overall comparison of intraoperative NRS scores among the 4 groups was statistically significant (*F* = 5.220, *P* = .002). Group 2 had the lowest intraoperative pain score, whereas group 1 had the highest. At 30 minutes postoperatively, the overall difference in NRS scores remained statistically significant (*F* = 11.019, *P* < .001), with group 2 again showing the lowest postoperative pain score (Table 2).

**Table 2:** Comparison of intraoperative and 30-min postoperative NRS pain scores among the 4 groups.

Group	n	Intraoperative NRS score	NRS score at 30 min postoperatively
Group 1	34	2.95 ± 1.46	2.22 ± 1.12
Group 2	37	1.75 ± 1.10	1.06 ± 0.73
Group 3	32	2.23 ± 1.31	1.43 ± 0.80
Group 4	39	2.12 ± 1.33	1.63 ± 0.78
*F* value		5.220	11.019
*P* value		.002	<.001

NRS = Numeric Rating Scale.

### 3.3. Interaction analysis of auricular therapy and anesthesia on postoperative pain

Further stratified analysis showed that among nonanesthetized patients, the auricular therapy group had a lower 30-minutes postoperative pain score than the nonauricular therapy group, with a mean difference of 1.161 points (95% confidence interval [CI]: 0.710–1.611; *P* < .001). Among locally anesthetized patients, the difference in postoperative pain scores between patients with and without auricular therapy was not statistically significant (*P* = .306). In the absence of auricular therapy, locally anesthetized patients had lower postoperative pain scores than nonanesthetized patients (mean difference, 0.787; 95% CI, 0.312–1.262; *P* = .002). Likewise, under the condition of concomitant auricular therapy, local anesthesia remained associated with lower postoperative pain scores (mean difference, −0.568; 95% CI, −0.914 to −0.223; *P* = .002) (Table 3).

**Table 3:** Interaction analysis of auricular therapy and anesthesia on postoperative pain in patients undergoing hysteroscopic examination.

Comparison dimension	Specific condition	Mean difference (95% CI)	*F* value	*P* value	Interpretation
Effect of auricular therapy	Among nonanesthetized patients	1.161 (0.710 to 1.611)	26.397	<.001	Significant
	Among locally anesthetized patients	−0.195 (−0.571 to 0.182)	1.065	.306	Not significant
Effect of anesthesia	Among patients without auricular therapy	0.787 (0.312 to 1.262)	10.955	.002	Significant
	Among patients with auricular therapy	−0.568 (−0.914 to −0.223)	10.741	.002	Significant

CI = confidence interval.

### 3.4. Comparison of postoperative 24-hours SAS scores among the 4 groups

One-way analysis of variance showed a statistically significant overall difference in postoperative 24-hours SAS scores among the 4 groups (*F* = 52.328, *P* < .001). Group 1 had the highest postoperative SAS score, whereas group 2 had the lowest. Pairwise comparisons showed that group 1 differed significantly from groups 2, 3, and 4 (all *P* < .001). No statistically significant difference was observed between groups 2 and 4 (*P* = .095) or between groups 4 and 3 (*P* = .436), whereas the difference between groups 2 and 3 reached statistical significance (*P* = .035) (Tables 4 and 5).

**Table 4:** Comparison of postoperative 24-hour SAS scores among the 4 groups.

Group	Description	n	SAS score	Minimum	Maximum
Group 1	Nonanesthesia without auricular therapy	34	29.32 ± 3.57	20	37
Group 2	Nonanesthesia with auricular therapy	37	22.38 ± 1.99	20	26
Group 3	Local anesthesia without auricular therapy	32	23.59 ± 2.60	20	32
Group 4	Local anesthesia with auricular therapy	39	23.15 ± 2.01	20	27

Overall comparison: *F* = 52.328, *P* < .001.

SAS = Self-Rating Anxiety Scale.

**Table 5:** Pairwise comparisons of postoperative 24-hour SAS scores among the 4 groups.

Comparison	Group names	*t* value	*P* value	Interpretation
Group 1 vs group 2	Nonanesthesia without auricular therapy vs nonanesthesia with auricular therapy	9.996	<.001	Significant
Group 1 vs group 4	Nonanesthesia without auricular therapy vs local anesthesia with auricular therapy	8.915	<.001	Significant
Group 1 vs group 3	Nonanesthesia without auricular therapy vs local anesthesia without auricular therapy	7.478	<.001	Significant
Group 2 vs group 4	Nonanesthesia with auricular therapy vs local anesthesia with auricular therapy	−1.690	.095	Not significant
Group 2 vs group 3	Nonanesthesia with auricular therapy vs local anesthesia without auricular therapy	−2.153	.035	Significant
Group 4 vs group 3	Local anesthesia with auricular therapy vs local anesthesia without auricular therapy	−0.784	.436	Not significant

### 3.5. Comparison of intraoperative vital signs and procedural indicators among the 4 groups

No statistically significant differences were observed among the 4 groups in procedure duration, intraoperative heart rate, systolic blood pressure, or diastolic blood pressure (all *P* > .05). However, hysteroscopy placement time differed significantly among groups (*P* < .001), with the longest placement time observed in group 1 and the shortest in group 3 (Table 6).

**Table 6:** Comparison of intraoperative vital signs and procedural indicators among the 4 groups.

Group	n	Procedure duration (min)	Hysteroscope placement time (s)	Intraoperative heart rate (beats/min)	Intraoperative systolic blood pressure (mm Hg)	Intraoperative diastolic blood pressure (mm Hg)
Group 1	34	11.84 ± 4.70	69.22 ± 39.88	76.13 ± 10.07	123.58 ± 13.87	77.30 ± 8.50
Group 2	37	13.96 ± 4.80	44.69 ± 21.55	80.36 ± 8.66	117.23 ± 10.45	74.26 ± 7.64
Group 3	32	12.79 ± 4.86	40.08 ± 17.32	78.05 ± 7.48	119.38 ± 10.82	77.56 ± 7.54
Group 4	39	11.66 ± 3.87	53.35 ± 27.21	79.21 ± 8.26	119.12 ± 9.53	73.37 ± 7.85
*P* value		.120	<.001	.210	.117	.056

### 3.6. Comparison of adverse event rates among the 4 groups

The adverse event rates in groups 1, 2, 3, and 4 were 2.9%, 13.5%, 18.8%, and 17.9%, respectively. The overall between-group difference was not statistically significant (χ^2^ = 4.696, *P* = .195). A total of 19 adverse events were recorded, all of which were mild and transient, and no severe adverse event occurred (Table 7).

**Table 7:** Comparison of adverse event rates among the 4 groups.

Group	Description	n	Patients with adverse events	Patients without adverse events	Adverse event rate (%)
Group 1	Non-anesthesia without auricular therapy	34	1	33	2.9
Group 2	Non-anesthesia with auricular therapy	37	5	32	13.5
Group 3	Local anesthesia without auricular therapy	32	6	26	18.8
Group 4	Local anesthesia with auricular therapy	39	7	32	17.9
Total	–	142	19	123	13.4

Overall comparison: χ^2^ = 4.696, *P* = .195.

### 3.7. Comparison of patient satisfaction among the 4 groups

All patients were rated as either “satisfied” or “very satisfied,” resulting in an overall satisfaction rate of 100.0%. Of the 142 patients, 116 (81.7%) were classified as very satisfied and 26 (18.3%) as satisfied. The distribution of satisfaction levels differed significantly among the 4 groups (χ^2^ = 10.834, *P* = .013), with group 2 showing the highest proportion of “very satisfied” responses (Table 8).

**Table 8:** Comparison of patient satisfaction among the 4 groups.

Group	Description	n	Very satisfied, n (%)	Satisfied, n (%)	Overall satisfaction (%)
Group 1	Non-anesthesia without auricular therapy	34	22 (64.7)	12 (35.3)	100.0
Group 2	Non-anesthesia with auricular therapy	37	35 (94.6)	2 (5.4)	100.0
Group 3	Local anesthesia without auricular therapy	32	27 (84.4)	5 (15.6)	100.0
Group 4	Local anesthesia with auricular therapy	39	32 (82.1)	7 (17.9)	100.0
Total	–	142	116 (81.7)	26 (18.3)	100.0

Overall comparison: χ^2^ = 10.834, *P* = .013.

## 4. Discussion

This study evaluated the associations of auricular therapy and local anesthesia with perioperative pain, postoperative anxiety, procedural indicators, adverse events, and satisfaction during hysteroscopic examination. The main findings were threefold. First, significant between-group differences were observed in both intraoperative and 30-minute postoperative NRS scores, and the nonanesthesia plus auricular therapy group had the lowest pain scores. Second, postoperative 24-hour SAS scores also differed significantly, with the nonanesthesia without auricular therapy group showing the highest anxiety burden and the nonanesthesia plus auricular therapy group the lowest. Third, adverse events were uncommon and mild, while overall satisfaction was high across groups and highest in the auricular therapy group without anesthesia. These findings suggest that auricular therapy may have practical value as an adjunctive strategy for symptom control during hysteroscopy, especially in patients managed without pharmacologic anesthesia. Current outpatient hysteroscopy guidance emphasizes minimizing pain and optimizing patient experience, and recent pain-management guidance for office uterine and cervical procedures similarly supports individualized, multimodal approaches rather than a single universal method.^[11]^

The pain findings are clinically relevant because pain remains one of the major barriers to successful office hysteroscopy. Recent reviews and guidelines consistently indicate that procedural discomfort can reduce procedure completion, impair tolerance, and negatively affect willingness to undergo repeat evaluation. They also emphasize that pain during hysteroscopy is multifactorial and influenced by cervical manipulation, uterine distension, patient anxiety, prior experience, and technical aspects such as entry technique and instrument characteristics. In that context, the lower pain scores observed in the auricular therapy group without anesthesia may indicate that this intervention is particularly useful when baseline analgesic support is limited.^[12,13]^ The interaction analysis further refines this interpretation. Among nonanesthetized patients, auricular therapy was associated with lower postoperative pain, whereas among locally anesthetized patients the additional association was not statistically significant. A reasonable explanation is that local anesthesia already reduced a substantial proportion of cervical and procedural pain, thereby narrowing the margin for an additional observable benefit from auricular therapy. This interpretation is consistent with recent guidance stating that analgesic effects in office gynecologic procedures are procedure-specific and that clinicians should be cautious in assuming that one modality will confer the same magnitude of benefit across different baseline analgesic conditions. It is also consistent with recent reviews showing that nonpharmacologic interventions may be helpful, but their measurable effect depends on the procedural context and comparator strategy.^[14]^

The current results also align with contemporary evidence that hysteroscopic pain is stage-dependent. A 2024 study evaluating pain across procedural steps under local anesthesia reported that pain was not evenly distributed across hysteroscopy and was particularly influenced by cervical passage and uterine entry. This stage-specific pattern helps explain why a supportive intervention may show benefit during and shortly after the procedure without necessarily altering all physiologic measures. It also supports the interpretation that an intervention acting on both sensory perception and procedural distress may improve patient-reported outcomes even when the procedure is relatively brief.^[15,16]^ The anxiety findings are similarly important. Group 1 had the highest postoperative SAS score, whereas group 2 had the lowest, and the differences between group 1 and the other groups were statistically significant. This pattern supports the concept that pain and anxiety during hysteroscopy are closely linked. Recent meta-analytic evidence on virtual reality during office hysteroscopy has shown that although pain reduction may be inconsistent across studies, anxiety reduction is more reproducibly observed. A 2024 meta-analysis concluded that virtual reality did not clearly decrease pain during office hysteroscopy but did reduce anxiety, whereas a more recent randomized trial in 2025 suggested that immersive distraction may improve both pain and stress outcomes in selected settings. Although auricular therapy is a different intervention, these studies support the broader principle that nonpharmacologic modulation of procedural distress can improve the patient experience even when direct analgesic effects vary.^[4,17]^

Several mechanisms may plausibly account for the observed associations. From a physiologic perspective, auricular stimulation has been proposed to influence nociceptive processing and autonomic regulation through auricular neural pathways, while from a clinical perspective it also functions as a structured supportive intervention involving repeated contact, guided attention, and reassurance. Recent perioperative evidence on auricular acupuncture indicates that ear-based interventions may reduce pain intensity in some surgical contexts, although the certainty of evidence remains limited by heterogeneity and methodological variation. Accordingly, the present findings should not be interpreted as proof of a specific mechanistic pathway, but rather as compatible with a combined sensory, autonomic, and supportive-care effect. The absence of significant between-group differences in heart rate and blood pressure deserves comment. One interpretation is that standard hemodynamic indicators were insufficiently sensitive to capture modest differences in perceived discomfort during a short outpatient procedure. Another is that the overall procedural burden remained within a generally tolerable range for most participants. By contrast, hysteroscope placement time differed significantly among groups, with shorter times in the anesthetized groups. This is clinically plausible, because local anesthesia may facilitate cervical manipulation and reduce resistance during uterine entry. Recent office hysteroscopy guidance emphasizes that procedural technique, operator experience, and technical facilitation all contribute to pain perception and procedural efficiency. The present results therefore support the view that patient comfort and technical performance are interrelated rather than independent dimensions of hysteroscopic care.^[18,19]^ The safety profile in this study was acceptable. Although the numerical adverse event rates were somewhat higher in the local anesthesia groups, the overall between-group difference was not statistically significant, and all events were mild and transient. This pattern is broadly consistent with recent guidance describing office hysteroscopy as a generally safe procedure when appropriate selection, counseling, and technique are used. Importantly, satisfaction was high in all groups, and the highest proportion of “very satisfied” responses was seen in the nonanesthesia plus auricular therapy group. Because satisfaction reflects not only pain relief but also perceived support, tolerability, and overall procedural acceptability, these findings suggest that the clinical value of auricular therapy may extend beyond pain intensity alone.^[20]^

In addition to the variables analyzed in this study, several clinical and procedural factors may influence pain and anxiety during hysteroscopy, including cervical stenosis, prior hysteroscopic experience, baseline psychological status, preprocedural anxiety level, examination indications, types of intrauterine lesions, and operator experience. These factors may affect procedural difficulty, pain perception, and emotional responses. Because complete information on these variables was not consistently available in the retrospective records, they could not be included in the current analysis and therefore represent potential sources of residual confounding.

Several limitations of this study should be acknowledged. First, this was a single-center retrospective comparative study, and the inherent limitations of retrospective analyses, including selection bias and unmeasured confounding, cannot be fully excluded. Second, although the sample size was moderate, the study population was derived from a single institution, which may limit the generalizability of the findings to other clinical settings and patient populations. Third, the analysis focused primarily on short-term perioperative outcomes, including pain during the procedure, pain at 30 minutes postoperatively, and anxiety at 24 hours after the examination; therefore, the longer-term effects of auricular therapy on symptom relief and patient experience remain unclear. Fourth, several potentially relevant clinical and procedural factors, including cervical stenosis, previous hysteroscopy experience, baseline anxiety status, operator experience, examination indications, and types of uterine cavity lesions, were not systematically recorded or adjusted for. Consequently, residual confounding may still exist despite the generally comparable baseline characteristics among groups. Finally, because of the observational design, the present findings should be interpreted as associations rather than evidence of causality. Future multicenter prospective studies with larger sample sizes, more comprehensive covariate adjustment, and longer follow-up are needed to further clarify the role of auricular therapy in perioperative symptom management during hysteroscopic examination.

## 5. Conclusion

In conclusion, this study suggests that auricular therapy may be associated with lower perioperative pain, lower postoperative anxiety, and higher satisfaction during hysteroscopic examination, with the clearest pain-related benefit observed in the nonanesthesia setting. Local anesthesia remained associated with lower postoperative pain and shorter hysteroscope placement time, but the incremental contribution of auricular therapy appeared less evident once local anesthesia had already been administered. Overall, these findings support further evaluation of auricular therapy as one component of an individualized, multimodal strategy for improving patient experience during office hysteroscopy.

## Author contributions

**Conceptualization:** Weizhu Zhu, Anqi Zhou, Xianwen Jin.

**Data curation:** Weizhu Zhu, Anqi Zhou, Xianwen Jin.

**Formal analysis:** Weizhu Zhu, Anqi Zhou, Xianwen Jin.

**Funding acquisition:** Weizhu Zhu.

**Investigation:** Weizhu Zhu.

**Writing – original draft:** Weizhu Zhu.

**Writing – review & editing:** Weizhu Zhu.
